# Identification of Myths and Misinformation About Treatment for Opioid Use Disorder on Social Media: Infodemiology Study

**DOI:** 10.2196/44726

**Published:** 2024-02-23

**Authors:** Mai ElSherief, Steven Sumner, Vikram Krishnasamy, Christopher Jones, Royal Law, Akadia Kacha-Ochana, Lyna Schieber, Munmun De Choudhury

**Affiliations:** 1 Khoury College of Computer Sciences Northeastern University Boston, MA United States; 2 Centers for Disease Control and Prevention Atlanta, GA United States; 3 Georgia Institute of Technology Atlanta, GA United States

**Keywords:** addiction treatment, machine learning, misinformation, natural language processing, opioid use disorder, social media, substance use

## Abstract

**Background:**

Health misinformation and myths about treatment for opioid use disorder (OUD) are present on social media and contribute to challenges in preventing drug overdose deaths. However, no systematic, quantitative methodology exists to identify what types of misinformation are being shared and discussed.

**Objective:**

We developed a multistage analytic pipeline to assess social media posts from Twitter (subsequently rebranded as X), YouTube, Reddit, and Drugs-Forum for the presence of health misinformation about treatment for OUD.

**Methods:**

Our approach first used document embeddings to identify potential new statements of misinformation from known myths. These statements were grouped into themes using hierarchical agglomerative clustering, and public health experts then reviewed the results for misinformation.

**Results:**

We collected a total of 19,953,599 posts discussing opioid-related content across the aforementioned platforms. Our multistage analytic pipeline identified 7 main clusters or discussion themes. Among a high-yield data set of posts (n=303) for further public health expert review, these included discussion about potential treatments for OUD (90/303, 29.8%), the nature of addiction (68/303, 22.5%), pharmacologic properties of substances (52/303, 16.9%), injection drug use (36/303, 11.9%), pain and opioids (28/303, 9.3%), physical dependence of medications (22/303, 7.2%), and tramadol use (7/303, 2.3%). A public health expert review of the content within each cluster identified the presence of misinformation and myths beyond those used as seed myths to initialize the algorithm.

**Conclusions:**

Identifying and addressing misinformation through appropriate communication strategies could be an increasingly important component of preventing overdose deaths. To further this goal, we developed and tested an approach to aid in the identification of myths and misinformation about OUD from large-scale social media content.

## Introduction

In the United States, more than 100,000 drug overdose deaths occurred in 2021 [1]. Beyond lives lost, the economic costs of both fatal opioid overdose and opioid use disorder (OUD) are estimated to be greater than 1 trillion US dollars per year [2,3]. Furthermore, the extent of OUD is significant; in 2020, about 2.7 million people in the United States aged 12 years or older met the diagnostic criteria for an OUD in the past year [4].

The American Psychiatric Association’s *Diagnostic and Statistical Manual of Mental Disorders, Fifth Edition* (DSM-5) defines OUD as a “problematic pattern of opioid use leading to clinically significant impairment or distress” [5]. OUD is characterized by 11 defining criteria, including taking opioids in larger amounts or over a longer period of time than intended [5]. Medications for opioid use disorder (MOUD), including methadone, buprenorphine, and extended-release naltrexone, are effective treatments for OUD [6]. MOUD increase treatment retention and reduce opioid use and overdose mortality, among other public health benefits [7]. Yet in 2020, it is estimated that only 11.2% of people with OUD received treatment with MOUD in the past year, based on data from the National Survey on Drug Use and Health [8]. Despite its effectiveness, MOUD are underused, in part due to stigma, financial constraints, treatment availability, and a lack of perceived treatment need [4].

When individuals do seek information on MOUD—often on the internet or through social media—they may access inaccurate and potentially harmful health misinformation [9]. A recent review pointed to several areas of misinformation regarding MOUD, including that MOUD may be detrimental to health and can be perceived as simply substituting one addiction with another [10]. Although social media provides a venue for individuals to seek help, advice, and support surrounding their OUD experiences, journeys, and recovery goals [11-13], these platforms also provide a mechanism for misinformation about MOUD to spread [14]. However, most studies to date have focused on general opinions and attitudes regarding MOUD, and less is known about specific misinformation that is shared. For example, Tofighi et al [15] conducted a qualitative analysis of 1010 Twitter (subsequently rebranded as X) posts related to MOUD, assessing general experiences and perceptions. A subsequent study by Chenworth et al [16] assessed tweets that mentioned methadone or suboxone and found that a large percentage expressed negative sentiment about MOUD. Pertaining specifically to misinformation, a study [14] quantified the prevalence of a single myth about MOUD across multiple social media and web-based communication platforms. This myth was drawn from clinical literature; however, it is likely that other myths or misinformation related to MOUD are emerging or being discussed that have not been previously described. Indeed, health care professionals’ understanding of what misinformation and myths may be circulating related to substance use disorder treatment is currently limited to expert opinion, and there is no systematic or large-scale quantitative approach to identify new opioid-related myths from web-based communications and social media platforms. Thus, in this study, we developed and evaluated an approach for identifying potentially novel myths that may exist regarding MOUD. This approach can help identify harmful content to inform strategies to educate clinicians and the public about MOUD and counter myths and misinformation related to MOUD.

## Methods

### Overview

**Figure 1:**
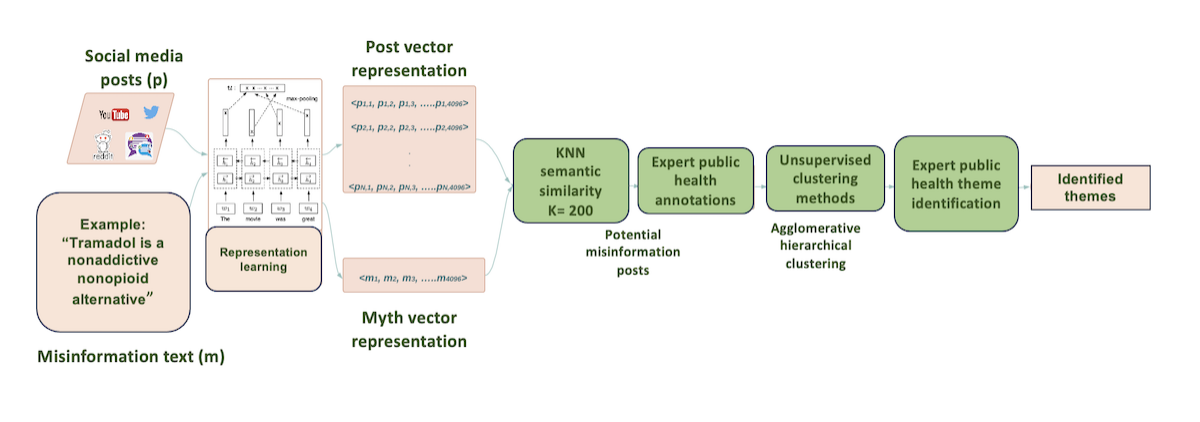
Pipeline for the identification of new myths pertaining to treatment for opioid use disorder. We start by transforming each post and myth into a mathematical representation. Next, we identify the posts closest to myths in meaning and have public health experts annotate them for accuracy. We then computationally cluster similar themes together and have public health experts assign a clinical theme. KNN: k-nearest neighbor.

To accomplish our goal of identifying potentially new myths about MOUD from social media, we used a multistep analytic pipeline (Figure 1), described in detail below. The steps included curating a data set of social media posts across multiple platforms; extracting posts with a high probability of including a myth; using a clustering algorithm to group these posts into themes; and lastly, examining the resulting content for language indicative of misinformation and myths.

### Data Set Curation

We first developed a lexicon of opioid-related keywords; for this, we adopted a 2-pronged approach that combined insights from the substance use literature and feedback from the substance use expert coauthors of this study. Our lexicon encompassed different types of opioids, such as natural opiates, semisynthetic opioids, and synthetic opioids, and included opioids that were prescription or illicit. For each generic drug name, we also included brand and combination product names. In addition, we also included street names of substances, where useful, from the Drug Enforcement Administration. Our final lexicon of 152 keywords was then used in the ensuing data collection.

Using this lexicon, we constructed a diverse data set from Twitter, YouTube, and online health communities (OHCs) such as Reddit and Drugs-Forum. For all the platforms we investigated, we focused on public posts and messages created between January 1, 2018, and December 31, 2019. Our data set collection methodology for Twitter included querying for all public posts that contained 1 of the words in our lexicon using the then-available Twitter Academic application programming interface (API). This process yielded 6,365,245 posts. For YouTube, due to rate limitations imposed in the data collection process by the platform’s API, we restricted the keywords to 11 MOUD treatment keywords such as buprenorphine and naltrexone. We used the YouTube API to identify 552 public YouTube videos that contain 1 of the 11 keywords in the title and then collected all of the associated comments (99,386 comments). We relied on expert domain knowledge to identify subforums pertinent to OUD for Reddit and Drugs-Forum. We used data from 22 opioid-specific subreddits (carfentanil, opiates, fentanyl, opiatesmemorial, modquittingkratom, methadone, suboxone, kratom, heroin, quittingkratom, Tianeptine, loperamide, naltrexone, oxycodone, OpiatesRecovery, opiatewithdrawal, lean, heroinaddiction, HeroinHeroines, OpiateChurch, suboxone, OurOverUsedVeins), resulting in 1,189,590 posts and 12,293,829 comments. Additionally, we collected 5549 messages posted under the various “Opiates and Opioids” subforums on Drugs-Forum. Throughout this paper, we combine Reddit and Drugs-Forum content under the category of OHCs because of their similar affordances.

### Mixed Methods Approach to Identify Social Media Posts Relevant to MOUD Myths

We began our analytic and data processing efforts by investigating three “seed“ myths drawn from the substance use literature [17-20]: (1) agonist therapy or medication-assisted treatment for OUD replaces one drug with another, (2) only patients treated with opioids who have certain characteristics are at higher risk for opioid addiction, and (3) tramadol is a nonaddictive nonopioid alternative. These seed myths were used, in concert with machine learning approaches, to filter the large volume of semantically rich social media content that would be subsequently investigated for the presence or absence of a new MOUD myth.

Specifically, we used InferSent, a sentence embedding method that provides semantic sentence representations [21], to construct document embeddings for the 3 myth statements noted above. Document embeddings are long sequences of numbers that mathematically represent the semantic meaning of each document (ie, a social media post). InferSent embeddings have been shown to outperform unsupervised methods such as SkipThought vectors on a range of natural language processing (NLP) tasks [21]. In our experiments, we evaluated the embedding values of the social media posts most similar to the seed myths and observed that, indeed, the most similar posts express a similar meaning but are expressed differently (eg, the following blockquote from a sample post in our data set was found to be similar to the seed myth “agonist therapy or medication-assisted treatment for OUD replaces one drug with another”).

....So I have decided to discontinue treatment. My family doesn't agree with this form of treatment and I'm not getting any support from them being on MMT. They don't see it any different than me doing heroin every day….

After constructing document embeddings for the seed myth text, we constructed document embeddings for all social media posts in our data set. Using the mathematical representation of each post, we were able to identify posts similar to the seed myths and containing additional information useful for understanding MOUD myths. Specifically, we identified the 200 most semantically similar posts per platform for each seed myth using the k-nearest neighbor (KNN) algorithm [22], implemented in Python’s *scikit-learn* library [23]. The KNN algorithm assumes that similar things exist in close proximity. In other words, KNN uses the idea that similar things are near each other—in our case, it would compare how close posts are to the seed myths. This process provided us with candidate social media posts that were likely to be discussing MOUD myths or related topics. Additional details of our machine learning and NLP approaches are provided in Multimedia Appendix 1 [21,24-34].

Because not all posts identified through the methods described above may be directly discussing a MOUD myth and document embeddings could pick up some noise, we performed a second data processing step, in which we harnessed annotations from public health experts. The experts reviewed and evaluated whether each post in our now-filtered data set of 800 messages (200 per platform) was relevant to 1 of the seed myths, discussing a new myth, or neither. Specifically, a total of 3 public health experts (coauthors of this paper) reviewed each of the 800 social media posts to perform this qualitative assessment and reach a consensus on the topic discussed therein. Public health experts included 2 clinicians and 1 doctoral-level epidemiologist. The experts reviewed all posts and collectively came to a consensus across all posts in our data set. Our rationale for the qualitative annotation approach followed the guidelines given in the seminal research of McDonald et al [35]. The experts leveraged thematic coding, an iterative process that involves multiple coders developing, discussing, and refining codes through continual discussion. Our qualitative annotation, followed by consensus-building discussion, is situated in grounded theory [36,37].

This manual review resulted in a total of 303 posts identified as discussing potentially new myths. The pipeline for identifying these posts is outlined in Figure 1.

### Methods to Understand Discussions of New Myths Arising From the Seeds

We developed additional machine learning–based techniques to better characterize the content of the 303 annotated posts. We leveraged an unsupervised machine learning technique known as hierarchical clustering [24]. This approach provides a probabilistic mechanism to group items (social media posts) into categories (discussion themes). Multimedia Appendix 1 describes this unsupervised technique in greater detail.

Hierarchical clustering was used to construct 10 categories from the 303 posts that are potentially indicative of new myths. All posts per theme were then presented to the above public health experts to interpret and name the extracted themes. To give richer context to the experts and help with the generation of theme descriptors, we also provided the linguistic markers (n=1-grams or single words) present in the posts for each theme using a commonly used lexical analytic generative model known as Sparse Additive Generative Models (SAGE) of Text [25]. SAGE identifies distinguishing words in our topic themes, where the SAGE magnitude of a word signals the degree of its uniqueness. Previous work has shown that SAGE outperforms other topic models, such as latent Dirichlet allocation (LDA) models, by focusing on high-frequency terms with accurate counts, thus leading to learning more robust interpretable topics [25]. Using this information, the experts developed descriptions of each category and identified myths related to OUD. After careful inspection, the experts aggregated a few topically similar themes, with a final focus on seven themes.

### Ethical Considerations

This study is considered exempt research since there are no human participants involved. As such, the study proceeded without obtaining informed consent from social media users. Moreover, social media users who authored posts in the data set were not compensated because the data were publicly available. Social media posts are anonymized by not including usernames in our analysis. All examples given in this study are slightly paraphrased from different social media platforms to further protect user confidentiality. The potential use of these findings by malicious actors cannot be overstated. Motivated actors perpetrating myths and misinformation surrounding OUD could use machine learning and NLP approaches to target susceptible people with OUD and redirect them toward clinically unverified treatments, leading to misinformation exacerbation. Additionally, people with OUD are often stigmatized on multiple levels. Individuals with OUD are perceived as dangerous, of moral failure, and called “addicts” [38]. In light of these possible negative outcomes, although we provided links to all open-source libraries associated with our computational analyses, we have not shared the text data from different social media platforms to minimize the possible identification of OUD social media users.

## Results

Our analytics pipeline resulted in the identification of 7 clusters or discussion themes of social media posts related to MOUD. Textbox 1 shows paraphrased example posts that represent new myths or potentially harmful information identified from this data set, and Table 1 displays the salient keywords identified by SAGE for each category to help provide further context.

**Example posts from our data set (n=303) that represent inaccurate or harmful information identified by our human-machine mixed strategies. Themes (in bold font) are labeled by public health experts. The bullet points depict example posts from our data set for each identified type of misinformation. Posts are slightly paraphrased to prevent traceability and author identification.**

**Discussing addiction or addictiveness**

- Calling addiction a disease cheapens what I mean.
- Addiction is just made up in your mind.
- Buprenorphine is more addictive than opioids.
- Calling it a disease is just an excuse.
- Fentanyl is less addictive than marijuana.
- Withdrawal from buprenorphine is worse than heroin.

**Taking medication for addiction is not true recovery**

- You have to be strong. Willpower is the only realway to quit.
- Who cares if people are addicted to kratom, it’s just like coffee,
- it’s good for you.
- Ibogaine cures addiction big time.

**Alternative or nonrecommended treatment for addiction**

- The only people overdosing are those taking street heroin.
- People with chronic pain are physically dependent on opioids to function like a normal person.

**Table 1:** Top salient words identified using Sparse Additive Generative Models (SAGE) [25] per cluster (total number of posts, n=303). Higher scores in the second column indicate greater saliency.

Topic or myth	Frequency, n (%)	SAGE keywords (score)
Treatments for opioid use disorder	90 (29.8)	therapy (1.36), mat (1.33), primary (1.31), assisted (1.28), treatment (1.24), care (1.22), replacement (1.18), buprenorphine (1.17), medication (1.13), and methadone (1.06)
Exploring the nature of addiction	68 (22.5)	physical (1.93), difference (1.85), dependence (1.83), addiction (1.77), between say (1.68), disease (1.53), not (1.47), no (1.41), and treated (1.4)
Pain, opioid use, and addiction	28 (9.3)	ppl (2.31), please (2.31), severe (2.31), majority (2.31), treating (2.31), function patients (2.27), chronic (2.27), people (2.23), and addicted (2.21)
Use of tramadol	7 (2.3)	study (3.47), extended (3.47), 200 (3.47), 30 (3.47), studies (3.29), mg tramadol (2.82), ibogaine (2.74), safe (2.54), and hard (2.54)
Physical dependence on medications	22 (7.2)	they’re (2.8), physically (2.57), trading (2.57), you’re (2.48), dependent (2.26), less (2.26), become (2.23), side (2.02), addicted (2.02), and being (1.9)
Intravenous or injection drug use	36 (11.9)	hit (1.61), nerve (1.61), hands (1.61), veins (1.52), feet (1.46), shoot (1.46), inject (1.46), artery (1.46), foot (1.46), and tying (1.46)
Pharmacologic properties, effects, and addictiveness of substances	52 (16.9)	sub (1.74), acetaminophen (1.6), targin (1.60), addictive (1.57), codeine (1.37), sometimes (1.36), drug (1.34), seizure (1.32), medical (1.32), and release (1.32)

The most prevalent category described and discussed treatments for opioid use disorder (90/303, 29.8%). Posts described several Food and Drug Administration (FDA)–approved medications for OUD, including buprenorphine and methadone, and nonapproved treatments such as kratom. Additional posts reference a regulated supply of heroin to treat OUD, while other posts discuss the need for long-term use of buprenorphine or methadone. New myths present in this category perpetuated the notion that addiction is not a medical disease and discussed alternative therapies such as kratom.

The second category explored the nature of addiction (68/303, 22.5%). Posts discussed the definition of addiction and the distinction between dependence and addiction and debated whether addiction is a disease. Similar to the first category, misinformation found in this category included statements that addiction is not a medical disease and the promotion of alternative therapies, such as “Ibogaine cures addiction big time.”

The third category described the pharmacologic properties, effects, and addictiveness of substances (52/303, 16.9%). Posts included comments on how addictive substances were compared with each other (eg, nicotine vs heroin or fentanyl vs marijuana). Additional posts discussed drug metabolism and drug synthesis. Misinformation in this category included statements that “Buprenorphine is more addictive than opioids” and that “Fentanyl is less addictive than marijuana.”

The fourth category focused on intravenous or injection drug use (36/303, 11.9%). Posts covered drug injection techniques, advice, and the health consequences of such injections. Misinformation in this category included statements that “withdrawal from buprenorphine is worse than heroin” and that “willpower is the only way to quit.”

The fifth category centered on pain, opioid use, and addiction (28/303, 9.3%). Posts covered the use of opioids for chronic pain, the risk of addiction when using opioids for pain, and the stigma associated with using opioids for pain. Misinformation in this category included diverse statements such as “the only people overdosing are those taking illicit heroin,” “people with chronic pain are physically dependent on opioids to function like a normal person,” and “Who cares if people are addicted to kratom, it’s just like coffee, it’s good for you.”

The sixth category described physical dependence on medications (22/303, 7.2%). Posts commented on the distinction between dependence and addiction and specifically noted kratom as resulting in less addiction. Misinformation in this category included statements promoting alternate therapies.

Finally, the seventh category largely described tramadol use (7/303, 2.3%). Posts commented on tramadol dosing and administration. A discussion of the alternative therapy, ibogaine, was also present.

Each of the 7 categories included posts that contained some form of new misinformation that was not present in the initial seed myths we used to build our detection approach. Textbox 1 presents examples of misinformation or myth text. Further review of the misinformation resulted in classifying the misinformation into three themes, as shown in Textbox 1: (1) discussing addiction or addictiveness, (2) taking medication for addiction is not true recovery, and (3) alternative or nonrecommended treatments for addiction.

## Discussion

### Overview

The aim of this study was to develop and test a methodology for identifying new myths and misinformation related to MOUD. While health professionals are cognizant of the presence of misinformation and its effects on patient populations [39,40], there is currently no systematic approach for identifying potentially harmful information that individuals who use substances are actually exposed to and discussing. We developed a semiautomated pipeline that uses known myths as seed text and a sophisticated NLP approach to identify other misinformation that is circulating. The approach used a recent algorithm released by Facebook Research for measuring text similarity in social media postings [21] and then explored results through automated clustering and human expert review. We found that this approach identified new myths and forms of misinformation beyond those used as seed myths, suggesting that this approach may be useful in identifying new and emerging forms of potentially harmful information that may be circulating.

The posts we identified were grouped into 7 main themes related to MOUD, each revealing inaccuracies upon review by public health experts. The most common misinformation included statements endorsing alternative therapies such as kratom or ibogaine or discouraging medication use, favoring less effective abstinence-only approaches [41]. These are critical areas that require ongoing public health attention since alternative therapies such as kratom have been linked to fatal overdoses, and nonpharmacologic therapy for OUD is associated with higher rates of drug resumption and mortality. Our approach, using automatic data mining techniques, successfully flagged these types of misinformation, even uncovering subtler concerns that have not received sufficient attention, such as misinformed statements about the addictiveness of buprenorphine and unwarranted fears about buprenorphine withdrawal. These findings are significant as buprenorphine, a partial-opioid agonist, stands as one of the most effective treatments for OUD [7]. In light of the rising impact of health misinformation on patient populations, this study addresses the lack of tools for identifying potentially harmful information that spreads. We introduce an approach that quantitatively taps into extensive discussions about addiction across major communication platforms, contributing to misinformation identification. This is pivotal because, although patients seek health information from social media, it is also a breeding ground for false information dissemination [42], and there is a lack of systematic tools to assess health information related to addiction shared on these platforms [43]. Most closely related to this study is the work done by Sarker et al [13], Garett and Young [44], and Johnson et al [45]. Garett and Young [44] conducted a review on how inaccurate and false beliefs by both patients and providers can lead to stigma and serve as barriers to receiving appropriate treatment. This study comments on the consequences of 4 types of stigma, including structural stigma, public stigma, self-stigma, and stigma associated with treatment medications [44]. Similar to this study, Johnson et al [45] conducted a content analysis of 33 YouTube videos to identify and understand the lived experiences of parents and families impacted by the opioid crisis. In contrast to their work, we focused on social media posts and comments to identify new myths surrounding OUD. Most closely related to this study is Sarker et al [13], wherein the authors similarly leveraged NLP methods and built a classifier that identified whether a post’s language promoted 1 of the leading myths challenging addiction treatment: that the use of agonist therapy for MOUD is simply replacing one drug with another [13]. However, this study differs in the sense that it does not focus on 1 particularly known myth but rather on finding new pieces of misinformation.

To our knowledge, no studies report interventions directed at web-based misinformation on MOUD, yet lessons from analogous interventions during the COVID-19 pandemic are informative. One study of US-based Facebook users showed decreased distance traveled among those who viewed video messages from health professionals during the 2020 holiday season [46]. Another study found that journalistic fact-checks may be effective against COVID-19 misinformation [47]. Finally, a third study demonstrated the utility of accuracy nudges in addressing COVID-19 misinformation [48]. Taken together, these interventions suggest initial steps in building the evidence base for infodemic response across public health areas. Further study is needed to understand interventions that address MOUD-related misinformation.

The greatest strength of this study is that it is nonobtrusive and leverages a large-scale data set along with advances in machine learning and NLP to identify new pieces of misinformation that could exacerbate OUD health-related risks. Nevertheless, this study is subject to limitations. First, the qualitative assessment of myths and misinformation was conducted by only a limited number of health experts. As myths can be nuanced, further work should aim to codify definitions and guidance around opioid-related myths, particularly as this field of study grows. Second, while we demonstrate success at identifying misinformation beyond the seed myths we used to initialize the algorithm, the nature and scope of all misinformation related to MOUD are not known, and thus we are unable to assess the sensitivity of our approach to capturing all misinformation that may exist. Nevertheless, the ability to quantitatively and algorithmically identify misinformation related to addiction is still an important advancement. Third, although the approach we use harnesses machine learning and NLP techniques, a component of our pipeline still relies on public health expert review. While this necessitates some labor, human-in-the-loop designs are a leading framework for product development, have certain advantages, and can be particularly useful for complex areas such as health misinformation where expert judgment is required [14]. Finally, we acknowledge that there are a multitude of social media–based communication modalities, and this study is limited to those that are publicly available. It is possible that the nature of health misinformation may differ by platform, and further study of these differences is needed.

Our research demonstrates promise in identifying myths and misinformation related to treatment for OUD included in social media posts. With rapidly rising opioid overdose fatality rates, the initiation and adoption of MOUD are increasingly urgent to prevent additional loss of life. Attention by health and public health professionals to the health misinformation that may be affecting individual decisions related to OUD treatment engagement and retention can be a critical element in enhancing prevention.

### Conclusions

Health misinformation regarding treatment for OUD is prevalent, contributing to the challenges of preventing opioid-related overdoses. However, a systematic and quantitative methodology to identify this misinformation is lacking. In response, we developed a multistage analytic pipeline to analyze social media posts from platforms such as Twitter, YouTube, Reddit, and Drugs-Forum for OUD-related misinformation. Our methodology successfully identified 7 main clusters of misinformation related to MOUD. The most salient topics for these myths include treatments for OUD, the nature of addiction, as well as the pharmacologic properties, effects, and addictiveness of substances. Further understanding of the identified myths and continual monitoring of emerging myths are critical in the battle against the opioid overdose epidemic.

## Appendix

Multimedia Appendix 1 Machine learning and clustering approaches.

## Abbreviations

API: application programming interface
DSM-5: Diagnostic and Statistical Manual of Mental Disorders, Fifth Edition
FDA: Food and Drug Administration
KNN: k-nearest neighbor
LDA: latent Dirichlet allocation
MOUD: medications for opioid use disorder
NLP: natural language processing
OHC: online health community
OUD: opioid use disorder
SAGE: Sparse Additive Generative Models

## Data Availability

The data sets generated during and/or analyzed during this study are available from the corresponding author on reasonable request.

## References

[1] Hedegaard H, Miniño AM, Spencer MR, Warner M (2021). Drug overdose deaths in the United States, 1999-2020. NCHS Data Brief.

[2] Luo F, Li M, Florence C (2021). State-level economic costs of opioid use disorder and fatal opioid overdose—United States, 2017. MMWR Morb Mortal Wkly Rep.

[3] Florence C, Luo F, Rice K (2021). The economic burden of opioid use disorder and fatal opioid overdose in the United States, 2017. Drug Alcohol Depend.

[4] Madras BK, Ahmad NJ, Wen J, Sharfstein JS (2020). Improving access to evidence-based medical treatment for opioid use disorder: strategies to address key barriers within the treatment system. NAM Perspect.

[5] Opioid use disorder: preventing and treating. Centers for Disease Control and Prevention.

[6] Medications to treat opioid use disorder research report. National Institute on Drug Abuse.

[7] Leshner AI, Mancher M, National Academies of Sciences, Engineering, and Medicine, Health and Medicine Division, Committee on Medication-Assisted Treatment for Opioid Use Disorder, Board on Health Sciences Policy (2019). Medications for Opioid Use Disorder Save Lives.

[8] (2021). Key substance use and mental health indicators in the United States: results from the 2020 National Survey on Drug Use and Health. Substance Abuse and Mental Health Services Administration.

[9] Suarez-Lledo V, Alvarez-Galvez J (2021). Prevalence of health misinformation on social media: systematic review. J Med Internet Res.

[10] Volkow ND, Frieden TR, Hyde PS, Cha SS (2014). Medication-assisted therapies--tackling the opioid-overdose epidemic. N Engl J Med.

[11] Young SD, Koussa M, Lee SJ, Perez H, Gill N, Gelberg L, Heinzerling K (2018). Feasibility of a social media/online community support group intervention among chronic pain patients on opioid therapy. J Addict Dis.

[12] Chancellor S, Nitzburg G, Hu A, Zampieri F, De Choudhury M (2019). Discovering alternative treatments for opioid use recovery using social media.

[13] Sarker A, Gonzalez-Hernandez G, Ruan Y, Perrone J (2019). Machine learning and natural language processing for geolocation-centric monitoring and characterization of opioid-related social media chatter. JAMA Netw Open.

[14] ElSherief M, Sumner SA, Jones CM, Law RK, Kacha-Ochana A, Shieber L, Cordier L, Holton K, De Choudhury M (2021). Characterizing and identifying the prevalence of web-based misinformation relating to medication for opioid use disorder: machine learning approach. J Med Internet Res.

[15] Tofighi B, El Shahawy O, Segoshi A, Moreno KP, Badiei B, Sarker A, Krawczyk N (2021). Assessing perceptions about medications for opioid use disorder and naloxone on Twitter. J Addict Dis.

[16] Chenworth M, Perrone J, Love JS, Graves R, Hogg-Bremer W, Sarker A (2021). Methadone and suboxone mentions on Twitter: thematic and sentiment analysis. Clin Toxicol (Phila).

[17] Heimer R, Hawk K, Vermund SH (2019). Prevalent misconceptions about opioid use disorders in the United States produce failed policy and public health responses. Clin Infect Dis.

[18] Wakeman SE, Barnett ML (2018). Primary care and the opioid-overdose crisis—buprenorphine myths and realities. N Engl J Med.

[19] Grinspoon P (2021). 5 myths about using suboxone to treat opiate addiction. Harvard Health Publishing.

[20] Wardhan R, Chelly J (2017). Recent advances in acute pain management: understanding the mechanisms of acute pain, the prescription of opioids, and the role of multimodal pain therapy. F1000Res.

[21] Conneau A, Kiela D, Schwenk H, Barrault L, Bordes A (2017). Supervised learning of universal sentence representations from natural language inference data. https://aclanthology.org/D17-1070/.

[22] Cover T, Hart P (1967). Nearest neighbor pattern classification. IEEE Trans Inform Theory.

[23] Machine learning in Python. Scikit-learn.

[24] Day WHE, Edelsbrunner H (1984). Efficient algorithms for agglomerative hierarchical clustering methods. J Classif.

[25] Eisenstein J, Ahmed A (2011). Sparse additive generative models of text.

[26] Lossio-Ventura JA, Bian J (2018). An inside look at the opioid crisis over Twitter.

[27] Tibebu S, Chang VC, Drouin CA, Thompson W, Do MT (2018). At-a-glance—what can social media tell us about the opioid crisis in Canada?. Health Promot Chronic Dis Prev Can.

[28] Hu H, Phan N, Geller J, Vo H, Manasi B, Huang X, Di Lorio S, Dinh T, Chun SA, Chen X, Sen A, Li WW, Thai MT (2018). Deep self-taught learning for detecting drug abuse risk behavior in tweets. Computational Data and Social Networks: 7th International Conference, CSoNet 2018, Shanghai, China, December 18–20, 2018, Proceedings, LNCS 11280.

[29] Hochreiter S, Schmidhuber J (1997). Long short-term memory. Neural Comput.

[30] Conneau A, Kruszewski G, Lample G, Barrault L, Baroni M (2018). What you can cram into a single vector: Probing sentence embeddings for linguistic properties.

[31] Blei DM, Ng AY, Jordan MI (2003). Latent dirichlet allocation. J Mach Learn Res.

[32] sklearn.cluster.AgglomerativeClustering. Scikit-learn.

[33] Bouguettaya A, Yu Q, Liu X, Zhou X, Song A (2015). Efficient agglomerative hierarchical clustering. Expert Syst Appl.

[34] Ashenden SK (2021). The Era of Artificial Intelligence, Machine Learning, and Data Science in the Pharmaceutical Industry.

[35] McDonald N, Schoenebeck S, Forte A (2019). Reliability and inter-rater reliability in qualitative research: norms and guidelines for CSCW and HCI practice. Proc ACM Hum Comput Interact.

[36] Kathy C (2006). Constructing Grounded Theory: A Practical Guide Through Qualitative Analysis, 1st Edition.

[37] Muller MJ, Kogan S (2010). Grounded theory method in HCI and CSCW. ResearchGate.

[38] Yang LH, Grivel MM, Anderson B, Bailey GL, Opler M, Wong LY, Stein MD (2019). A new brief opioid stigma scale to assess perceived public attitudes and internalized stigma: evidence for construct validity. J Subst Abuse Treat.

[39] Kennedy-Hendricks A, Busch SH, McGinty EE, Bachhuber MA, Niederdeppe J, Gollust SE, Webster DW, Fiellin DA, Barry CL (2016). Primary care physicians' perspectives on the prescription opioid epidemic. Drug Alcohol Depend.

[40] van Boekel LC, Brouwers EPM, van Weeghel J, Garretsen HFL (2013). Stigma among health professionals towards patients with substance use disorders and its consequences for healthcare delivery: systematic review. Drug Alcohol Depend.

[41] Barber M, Gardner J, Savic M, Carter A (2020). Ibogaine therapy for addiction: consumer views from online fora. Int J Drug Policy.

[42] De Choudhury M, Morris MR, White RW (2014). Seeking and sharing health information online: comparing search engines and social media.

[43] Vosoughi S, Roy D, Aral S (2018). The spread of true and false news online. Science.

[44] Garett R, Young SD (2022). The role of misinformation and stigma in opioid use disorder treatment uptake. Subst Use Misuse.

[45] Johnson KF, Worth A, Brookover D (2019). Families facing the opioid crisis: content and frame analysis of YouTube videos. Fam J.

[46] Breza E, Stanford FC, Alsan M, Alsan B, Banerjee A, Chandrasekhar AG, Eichmeyer S, Glushko T, Goldsmith-Pinkham P, Holland K, Hoppe E, Karnani M, Liegl S, Loisel T, Ogbu-Nwobodo L, Olken BA, Torres C, Vautrey PL, Warner ET, Wootton S, Duflo E (2021). Effects of a large-scale social media advertising campaign on holiday travel and COVID-19 infections: a cluster randomized controlled trial. Nat Med.

[47] Pennycook G, McPhetres J, Zhang Y, Lu JG, Rand DG (2020). Fighting COVID-19 misinformation on social media: experimental evidence for a scalable accuracy-nudge intervention. Psychol Sci.

[48] Kreps SE, Kriner DL (2022). The COVID-19 infodemic and the efficacy of interventions intended to reduce misinformation. Public Opin Q.

