# Supplementary material for: Identification of Myths and Misinformation About Treatment for Opioid Use Disorder on Social Media: Infodemiology Study
Source: JMIR Form Res. 2024 Feb 23;8:e44726. doi: 10.2196/44726 (PMC10924265; doi:10.2196/44726)
Supplement: Multimedia Appendix 1 [file formative_v8i1e44726_app1.docx]

**Supplementary Materials**

**Machine Learning Approach for Identifying OUD Myth Posts**

Online discourse surrounding OUD is semantically rich; that is, there are different words and combinations of words people use to convey meaning. Previous literature has quantitatively and qualitatively investigated various categories of language pertaining to OUD, including opioid use (own use, use by others, co-use) and perception (commentary on opioid crisis or opioids in general) [38-40]. To identify posts relevant to the three myths under investigation from the huge search space, we first leveraged representation learning – a set of techniques that allows a system to automatically discover the representations needed for feature detection or classification from raw data [21], and then constructed document-level embeddings (consisting of 4096 dimensions) of the myth statement noted above. For this, we used a bidirectional LSTM (Long Term Short Term Memory) sentence encoder model universally trained on a natural language inference task [41]. LSTM is a suitable choice here since it allows us to learn long-term dependencies among words in sentence structures. We then used this model to encode all the seed myths and all the posts collected. There is no consensus on what is the best sentence/paragraph embedding method. Recent work has shown that some sentence embedding methods outperform others depending on the downstream task [42]. It has also been shown that InferSent is more adaptable and can achieve better results in transfer tasks compared to unsupervised methods like skip-thought vectors, FastSent, and sent2vec [21]. Additionally, we experimented with both BERT and InferSent embeddings and we found that when we used InferSent embeddings, we were able to retrieve more semantically relevant content related to the seed myths in comparison to BERT-based embeddings, demonstrating the generation of high-quality sentence representations suitable for our task.

Following this step, we obtained the *K*-Nearest Neighbor (KNN), where *K* = 200, semantically most similar posts per platform for each myth. The KNN algorithm is a non-parametric supervised learning method that learns patterns from the *K* closest training examples in a dataset and then outputs the closeness of a previously unseen object to the learned patterns by averaging the values of its *K* nearest neighbors. Our choice of K was intended to strike a balance between annotation resources and coverage for the most semantically similar posts to the seed myth.

**Clustering Approaches to Identify New Myth Discussion Themes**

We used two types of unsupervised machine learning techniques – topic modeling and hierarchical clustering to group the 306 new myths indicative posts into discussion themes. In the former set of techniques, we used an established method known as the Latent Dirichlet Allocation (LDA) [43]. LDA is basically a generative statistical model that allows sets of observations to be explained by unobserved groups that explain why some parts of the data are similar. For example, if observations are words collected into documents, it posits that each document is a mixture of a small number of topics and that each word’s presence is attributable to one of the document’s topics. To optimize interpretability, we used Point Mutual Information scoring to combine bigrams and trigrams in the 306 new myth posts, that are more likely to occur together rather than being independent.

For the hierarchical clustering approach, we used an Agglomerative method [24] that is based on a bottom-up mechanism (i.e., starting with small clusters and merging them together to create larger clusters). More elaborately, the algorithm starts by treating each observation as a separate cluster. Then, it repeatedly executes the following two steps: (1) identify the two clusters that are closest together, and (2) merge the two most similar clusters. This iterative process continues until all the clusters are merged together. Essentially, the endpoint is a set of clusters, where each cluster is distinct from each other cluster, and the objects within each cluster are broadly similar to each other. Hierarchical clustering was chosen as it is widely used, applicable to most data types, does not require a pre-defined parameter (e.g., *K* in K-means clustering), and presents a complete picture of the underlying structure [48, 49]. We leveraged Python’s scikit-learn library to tune the Agglomerative hierarchical clustering algorithm [44]. For this approach, we use the document-level embeddings (ref. Supplement ) as a way to represent each of the 306 new myth posts in the semantic space.

Our public health experts analyzed the results of the two approaches and concurred that, unsurprisingly, the hierarchical clustering approach that leverages semantic representation outperforms the LDA-based mechanism. This is due to the fact that LDA relies on simple surface lexical cues while the data fed to the Agglomerative mechanism contains deep semantic structures.

We leverage Sparse Additive Generative Models of Text (SAGE) to extract salient words related to each cluster, before and after expert annotation. SAGE aims to identify the most informative terms in a given body of text. It employs a sparse representation, focusing on a select set of crucial terms rather than the entire vocabulary. The model assumes that a document is a result of additive contributions from these important terms, which aids in interpretability. By emphasizing sparsity and key term selection, SAGE helps extract meaningful patterns and insights from large textual datasets [25].
